# Internal dose assessment of ^148^Gd using isotope ratios of gamma-emitting ^146^Gd or ^153^Gd in accidently released spallation target particles

**DOI:** 10.1038/s41598-020-77718-3

**Published:** 2020-12-14

**Authors:** C. Rääf, V. Barkauskas, K. Eriksson Stenström, C. Bernhardsson, H. B. L. Pettersson

**Affiliations:** 1 Medical Radiation Physics, Department of Translational Medicine, Malmö, Lund University, 205 02 Malmö, Sweden; 2 Division of Nuclear Physics, Department of Physics, Lund University, 221 00 Lund, Sweden; 3 Department of Radiation Physics, IMV, Faculty of Health Sciences, Linköping University, 581 85 Linköping, Sweden

**Keywords:** Applied physics, Nuclear physics, Techniques and instrumentation, Risk factors, Physics

## Abstract

The pure alpha emitter ^148^Gd may have a significant radiological impact in terms of internal dose to exposed humans in case of accidental releases from a spallation source using a tungsten target, such as the one to be used in the European Spallation Source (ESS). In this work we aim to present an approach to indirectly estimate the whole-body burden of ^148^Gd and the associated committed effective dose in exposed humans, by means of high-resolution gamma spectrometry of the gamma-emitting radiogadolinium isotopes ^146^Gd and ^153^Gd that are accompanied by ^148^Gd generated from the operation of the tungsten target. Theoretical minimum detectable whole-body activity (MDA) and associated internal doses from ^148^Gd are calculated using a combination of existing biokinetic models and recent computer simulation studies on the generated isotope ratios of ^146^Gd/^148^Gd and ^153^Gd/^148^Gd in the ESS target. Of the two gamma-emitting gadolinium isotopes, ^146^Gd is initially the most sensitive indicator of the presence of ^148^Gd if whole-body counting is performed within a month after the release, using the twin photo peaks of ^146^Gd centered at 115.4 keV (MDA < 1 Bq for ingested ^148^Gd, and < 25 Bq for inhaled ^148^Gd). The corresponding minimum detectable committed effective doses will be less than 1 µSv for ingested ^148^Gd, but substantially higher for inhaled ^148^Gd (up to 0.3 mSv), depending on operation time of the target prior to the release. However, a few months after an atmospheric release, ^153^Gd becomes a much more sensitive indicator of body burdens of ^148^Gd, with a minimum detectable committed effective doses ranging from 18 to 77 µSv for chronic ingestion and between 0.65 to 2.7 mSv for acute inhalation in connection to the release. The main issue with this indirect method for ^148^Gd internal dose estimation, is whether the primary photon peaks from ^146^ and ^153^Gd can be detected undisturbed. Preliminary simulations show that nuclides such as ^182^Ta may potentially create perturbations that could impair this evaluation method, and which impact needs to be further studied in future safety assessments of accidental target releases.

## Introduction

The European Spallation Source (ESS), located north-east of the city of Lund in south-western Sweden, is designed to be the most powerful neutron source in the world using a 5 MW proton beam irradiation against a tungsten target^1–3^. An inevitable side effect of neutron generation during spallation reactions is the production of various radionuclides in the spallation source. During a 5 years operation of the ESS tungsten target, it is estimated that a number of gamma emitters will be produced, e.g. ^187^ W (> 10^16^ Bq) and ^172^Hf (> 10^15^ Bq), as well the pure beta emitters such as ^3^H (~ 10^15^ Bq) and pure alpha emitters as ^148^Gd (> 10^14^ Bq)^2^. The national competent authority in Sweden regarding emergency preparedness (Swedish Radiation Safety Authority, SSM) commissioned ESS to elaborate potential technical scenarios that could lead to an atmospheric release of spallation source particles^3^. Of these scenarios SSM considered the one involving loss of cooling of the spallation source while the neutron production operates at full effect (5 MW), being the one that dimensions the local emergency planning zone. Within minutes of proton beam irradiation of the tungsten target, the temperature increase in the target will in this event cause a fraction of the tungsten to melt and oxidize. About 20 kg of this fraction, including evaporated contaminated moderator water, will then be released through the pressure relief system to the surrounding atmosphere at a stack height of 30 m altitude. An additional release of tungsten (< 0.2 kg) will occur more than 80 min later in this process when hydrogen deflagration will eject about 0.5% of the remaining melted and oxidized target material into the atmosphere. In this accident scenario, target particles containing radionuclides, such as ^172^Hf, ^182^Ta, and ^187^W, have been estimated to be of largest radiological importance in terms of external exposure of humans from ground deposition of release radionuclides^3–6^. However, in terms internal exposure by inhalation or ingestion through contaminated foodstuff, the single most important radionuclide in estimated release scenarios is the pure alpha emitter ^148^Gd (E_a_ = 3.2 meV, T_½_ = 74.6 y)^3^, due to its high dose coefficient of up to 3 × 10^–5^ Sv Bq^−1^, which is comparable to alpha emitters such as ^223^Ra and ^238^Pu^7^.

Rääf et al.^8^ estimated the minimum detectable intakes of the gamma emitting spallation source nuclides ^172^Hf, ^182^Ta, and ^187^W to be 0.26, 0.04, and 65 kBq, respectively, when measured 24 h post intake using a large (123% relative efficiency), high-resolution HPGe in vivo whole-body counter in a low-background environment. When considering the alpha emitting ^148^Gd this technique is not directly available for internal dose assessments. However, since production of ^148^Gd in the tungsten target is accompanied by other gadolinium isotopes that are gamma emitters, it has been suggested that the presence of ^148^Gd could be indirectly estimated by in gamma spectrometry provided that the isotope ratios of the gamma-emitting isotopes are known^3^. Potential isotopes for such assessment are ^146^Gd (T½ = 48.3 days) and ^153^Gd (T½ = 240.4 days). The three principal gadolinium isotopes generated in a tungsten target from proton irradiation are listed in Table 1 together with their respective physical half-time and decay mode^9^.

**Table 1:** Alpha and photon energies (keV) and associated emission probability, n_g_, of the emission lines of gadolinium isotopes generated from proton irradiation of a W target^9^.

Nuclide	T_½_ (d)	Decay mode	Predominant α- or γ-lines (keV; emission probability, *n*_*g*_)
^146^Gd	48.3	Electron capture, gamma to ^146^Eu (T_½_ = 4.61 days with principal gamma lines at 633.1 and 634.1 keV; sum of n_g_ = 0.809)	154.6 (0.47) 114.7 (0.441) 115.5 (0.441)
^153^Gd	240.4	Electron capture, gamma to ^153^Eu (stable)	97.4 (0.29) 103.2 (0.211)
^148^Gd	76.4	Alpha emission to ^144^Sm (stable)	3271.21 (1)

The aim of this study is to suggest a method of indirect whole-body gamma ray counting of ^148^Gd that could facilitate a rapid assessment of the internal doses to affected humans after dispersion of ^148^Gd to the environment. By combining theoretical isotope ratios of ^146^Gd/^148^Gd and ^153^Gd/^148^Gd in an irradiated W target of the ESS, based on simulations presented in a previous study by Barkauskas and Stenström^2^, with biokinetic relationships derived from models presented by the International Commission of Radiological Protection (ICRP), we aim to estimate the minimum detectable whole-body burdens and associated internal dose from the alpha emitter ^148^Gd for a high efficiency high-resolution whole-body counting set-up presented in detail by Rääf et al.^8^. No detailed modelling of the fate of gadolinium in the terrestrial environment outside of ESS has been published, although internal estimates exist, which use e.g. americium and lanthanum as a chemical analogues when applying values for transfer parameters used in terrestrial models defined by IAEA (2001)^10^. Current studies on the ecological behaviour of gadolinium in this environment is launched (Lund University, 2019^11^). In absence of explicit estimates on the ecological half-time of gadolinium in vital environmental compartments such as crops, pasture, garden products and fresh water, we have conservatively assumed the following: i) inhalation of airborne gadolinium occurs momentarily after the accident, ii) during the first year upon release, there will be a transfer of radioactive gadolinium via the food chain to man, resulting in a constant daily ingestion rate.

## Theoretical outline and methods

### Biokinetic model

ICRP has presented a systemic biokinetic model for gadolinium^10^. Like all rare earth metals, gadolinium has a low uptake into tissue when ingested. ICRP 141^12^ presents a systemic biokinetic model for Gd and proposes a very low gastrointestinal (GI) uptake fraction (f_1_) of 0.0005 based on literature surveys^13^. The internal doses incurred are hence estimated to be rather low in relation to the activity intake, compared with more easily incorporated radionuclides such as radiocaesium, which is associated with fission products released from nuclear accidents or nuclear weapons debris. However, given the average transit time of 36 h for foodstuffs through the GI tract (described by, e.g., ICRP 100^14^), intakes of gadolinium by humans must still be considered, as the gadolinium isotopes may cause internal exposure during its passage time as well. In case of accidental releases from the tungsten target, the likely physiochemical form would be as particles, volatilized tungsten, and tungsten oxides^15,16^. When using that model, there appears to be a long time (< 1 y) before reaching the equilibrium whole-body stable gadolinium level at chronic intake. An ingestion of 1 Bq day^−1^ of any of the listed gadolinium isotopes listed in Table 1 corresponds to an infusion of 0.0005 Bq/day to systemic tissues. At 1 year after onset of the chronic intake of 1 Bq day^−1^ of a given gadolinium isotope, the systemic gadolinium content in a human will be 0.125 Bq, 0.025 Bq, and 0.075 Bq for ^148^Gd, ^146^Gd, and ^153^Gd, respectively. Moreover, it is estimated that equilibrium in the whole-body content of stable gadolinium is not reached until after 30 years of constant intake. For ^148^Gd, the equilibrium level is then estimated to be approximately 0.9 Bq per 1 Bq daily ingestion.

For systemically incorporated Gd, a large fraction will be found in soft tissue (approximately 17.3%). However, a yet larger fraction (55.1%) will be found in the cortical bone surface. This will result in a nearly homogeneous distribution in the whole body. However, due to the extremely low GI uptake (f_1_ = 0.0005 according to ICRP 141^12^), it is not anticipated that the component of systemic uptake of gadolinium will be important in comparison to the fraction of gadolinium in the GI tract. Hence, for in vivo whole-body counting of gamma emitting gadolinium isotopes in subjects who have chronically ingested radiogadolinium, a measurement geometry assuming major uptake in the abdominal region is more appropriate (See section “Estimating ^148^Gd whole-body burden and cumulative committed effective dose by means of high-resolution gamma spectrometry”).

### Relating ^148^Gd body burden with activity ratios of released gamma emitting gadolinium isotopes

**Figure 1:**
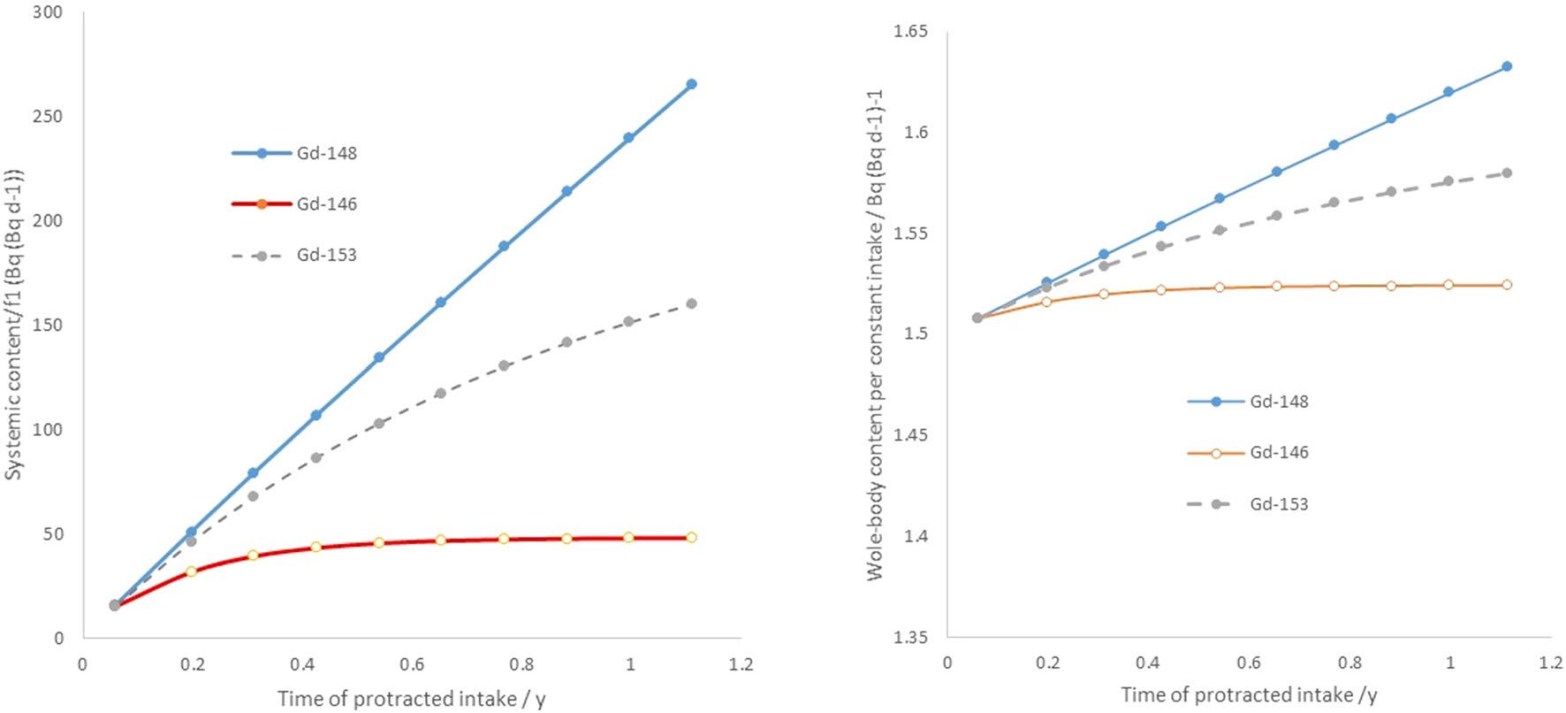
Left: Build-up of systemic ^146^Gd, ^148^Gd and ^153^Gd in human tissue after a daily intake of 1 Bq day^−1^ per isotope, normalised to uptake fraction f_1_(= 0.0005). Right: Build-up of whole-body activity (sum of systemic and GI contents) for ^146,148,153^Gd normalized per daily intake of each isotope, q_146,148,153_(t).

Some model derivations are needed to yield expressions that relate what is measurable by means of whole-body counting in a scenario with widespread release of radioactive gadolinium with a certain distribution between released gadolinium isotopes, ^146^Gd, ^148^Gd and ^153^Gd, to incurred committed effective dose of exposed subjects from ^148^Gd. Considering a passage time (residence time) in the GI tract of 36 h^14^, a chronic ingestion of 1 Bq/day of a given gadolinium isotope will lead to an equilibrium level of 1.5 Bq per Bq/day intake in the GI tract, if disregarding the low fraction (f_1_ = 0.0005) that has been taken up systemically. Given the long physical half-life of ^148^Gd (T½ = 76.4 y), this means that, after 1 year of chronic constant intake of ^148^Gd, the expected gadolinium abundance in the GI content during passage will be more than one order of magnitude larger than the fraction of ^148^Gd being incorporated systemically. Thus, in practice for protracted internal exposures, radioactive gadolinium will predominantly be found in the abdominal part of the body (Fig. 1, Right). It will also mean that, even for very long protracted intakes, the systemic gadolinium will only be a small fraction of the whole-body burden at any given time after the onset of the intake.

For short-lived gadolinium isotopes, the colon doses are more relevant over the long term compared with the dose to systemic tissue. The whole-body activity of a given gadolinium isotope, Q_Gd_, at any given time is the sum of the component in the GI contents, Q_Gd,GIcont_(t), and the systemically incorporated Gd, Q_Gdsys_(t):

1 $$Q_{Gd} \left( {\text{t}} \right) = { }Q_{d,GIcont} G\left( {\text{t}} \right){ } + { }Q_{Gd,sys} \left( {\text{t}} \right)$$

Using^14^ for the systemic component and a 36 h retention time of the inert fraction of gadolinium in the GI tract, the gadolinium content as a function of time after a constant protracted daily intake, I_Gd_ (Bq day^−1^), can be expressed in terms of intake normalized body content, q_Gd_(t):

2 $$q_{Gd} \left( {\text{t}} \right) = \frac{{Q_{Gd} \left( t \right)}}{{I_{Gd} }}{ } = \left( {\frac{{Q_{Gd,GIcont} \left( {\text{t}} \right){ } + { }Q_{Gd,sys} \left( t \right)}}{{I_{Gd} }}} \right) = Q_{Gd,GIcont} \left( t \right) \cdot h_{Gd} \left( t \right) + { }Q_{Gd,sys} \left( t \right) \cdot m_{Gd} \left( t \right)$$

contents and systemic tissue, respectively, normalized against the daily protracted intake I_Gd_. For ^148^Gd, the normalized body content q_Gd-148_(t) as a function of time, t (d), after start of chronic ingestion of 1 Bq day^−1^, can be expressed as follows:

3 $$\begin{array}{*{20}l} {q_{Gd - 148} \left( {\text{t}} \right) = {1} \cdot {\text{t}} ;} \hfill & {{\text{if}}\;{\text{ t}} < {1}.{\text{5 d}}} \hfill \\ {1.5 + 0.93 \cdot \left( {1 + e^{{ - \frac{ln2}{{1821}} \cdot t}} } \right);} \hfill & {{\text{if}}\;{\text{ t}} \ge {1}.{\text{5 d}}} \hfill \\ \end{array}$$

Equation 3 is obtained by curve regression from the combination of the systemic biokinetic model in^12^ and the ICRP colon model described in^14^. In the right from of Fig. 1 the plot of Eq. (3) is given for ^146^Gd, ^148^Gd and ^153^Gd, respectively.

The whole-body activity of the gamma-emitting gadolinium isotopes ^146^Gd and ^153^Gd at a given time t after the onset of the chronic intake of ^148^Gd, I_Gd-148_, can be related to the whole-body activity of the alpha emitter ^148^Gd with the corresponding retention of the accompanied gamma-emitting gadolinium isotopes ^146^Gd and ^153^Gd. Thus, for a scenario of intakes from a release containing a composition of different radio-gadolinium isotopes, the whole-body activity of ^148^Gd, Q_Gd-148_, can be expressed in terms of the corresponding whole-body activity of either of the gamma-emitting radionuclides ^146^Gd or ^153^Gd, using the following relationships:

4 $$Q_{d - 148} G\left( t \right) = { }Q_{Gd - 146,GIcont} \left( t \right) \cdot h_{148/146} \left( t \right){ } + { }Q_{Gd - 146,syst} \left( t \right) \cdot m_{148/146} \left( t \right) = k_{148/146} \left( t \right) \cdot Q_{Gd - 146} \left( t \right)$$

or

5 $$Q_{Gd - 148} \left( t \right) = { }Q_{Gd - 153,GIcont} \left( t \right) \cdot h_{148/153} \left( t \right){ } + { }Q_{Gd - 153,syst} \left( t \right) \cdot m_{148/153} \left( t \right) = { }k_{148/153} \left( t \right) \cdot Q_{Gd - 153} \left( t \right)$$

where h_148/146_(t) is the time-dependent activity ratio between ^146^ and ^148^Gd in the GI contents divided by the daily intake of ^148^Gd, I_Gd-148_ (Bq day^−1^), and h_153/146_(t) is the corresponding activity ratio for ^153^Gd and ^148^Gd. Moreover, in analogy with the expression in Eq. (2), the term m_148/146_(t) in Eq. (4) is the activity ratio at time t between ^146^ and ^148^Gd, divided by I_Gd-148_ in the systemic tissues, and m_153/146_(t) is the corresponding ratio for ^153^Gd and ^148^Gd. In turn, the expressions in Eqs. (3) and (4) can be rewritten as a time-dependent relationship between the whole-body burden of Q_Gd-148_ and Q_Gd-146_ and Q_Gd-153_, respectively, in terms of the time-dependent scaling factors k_148/146_ and k_148/153_, respectively. The purpose of the expressions in Eqs. (3) and (4) is thus to relate the whole-body burden of the alpha-emitting ^148^Gd with quantities Q_Gd-148_ and Q_Gd-153_, which are measurable by means of whole-body counting.

Since for a chronic ingestion of radiogadolinium Q_Gi-cont_ >  > Q_syst_, Eqs. (3) and (4) can virtually be rewritten as

6 $$Q_{Gd - 148} \left( t \right) = { }Q_{Gd - 146,GIcont} \left( t \right) \cdot h_{148/146} \left( t \right)){ }\sim { }k_{148/146} \left( t \right) \cdot Q_{Gd - 146} \left( t \right)$$

or

7 $$Q_{Gd - 148} \left( t \right) = { }Q_{Gd - 153,GIcont} \left( t \right) \cdot h_{148/153} \left( t \right)){ }\sim { }k_{148/153} \left( t \right) \cdot Q_{Gd - 153} \left( t \right)$$

The factors k_148/146_(t) and k_148/153_(t) are in turn given by the initial activity proportions at the start of the intake (i.e., at time t = 0). If the initial activity ratio between ^148^ and ^146^Gd is denoted as a_146/148_, and the ratio between ^148^ and ^153^Gd is denoted as a_153/148_, then the values of h_148/146_(t_0_) and m_148/146_(t_0_) will be equal to 1/a_146/148_, and the values of h_148/153_(t0) and m_148/153_(t_0_) will be equal to 1/a_153/148_. Likewise, the factors k_148/146_(t) and k_148/153_(t) will then be equal to 1/a_146/148_ and 1/a_153/148_, respectively, at t = t_0_. In this study, a_146/148_ and a_153/148_ are simulated using the FLUKA code^17,18^, where t_0_ = time of the release to the environment. Assuming equal biochemical and biokinetic behavior for all radiogadolinium isotopes, the daily intake of ^146^Gd and ^153^Gd will then be I_Gd-148_/a_148/146_ and I_Gd-148_/a_148/153_, respectively. In this computational study, no account of ecological turnover of dispersed gadolinium was considered, meaning that the effective ecological half-times of gadolinium isotopes are assumed to be equal to their corresponding physical half-lives.

### Committed effective dose calculations

ICRP^7^ provides data on committed effective dose coefficients per unit intake of gadolinium isotopes. The committed effective dose, E_Gd-148_ (mSv), incurred at time t (d) after the start of a chronic intake of ^148^Gd, denoted as I_Gd-148_ (Bq day^−1^), can then be expressed as:

8 $$E_{Gd - 148} \left( t \right) = I_{Gd - 148} \cdot t \cdot e_{Gd - 148}$$

where e_Gd-148_ (mSv Bq^−1^) is the committed effective dose coefficient taken from ICRP 119^7^ for an adult person. The coefficient refers to the time-integrated effective dose incurred upon intake (ingestion or inhalation) of a radionuclide. For the alpha emitter, this coefficient is 5.5·10^–5^ mSv Bq^−1^ for ingestion of ^148^Gd, which is more than 50 times higher than for ingestion of ^146^Gd and 200 times higher than for ingestion of ^153^Gd. The corresponding formulae for ^146^Gd and ^153^Gd are

9 $$E_{Gd - 146} = I_{Gd - 146} \cdot t \cdot e_{Gd - 146} = { }I_{Gd - 148} \cdot a_{146/148} \left( t \right) \cdot t \cdot e_{Gd - 146} = { }I_{Gd - 153} \cdot a_{146/153} \left( t \right) \cdot t \cdot e_{Gd - 146}$$

Exploiting that I_Gd-148_ is equal to the ratio Q_Gd-148_(t)/q_Gd-148_(t), Eq. (8) can be expressed as

10 $$E_{Gd - 148} \left( t \right) = \left( {Q_{Gd - 148} \left( t \right)/q_{Gd - 148} \left( t \right)} \right) \cdot t \cdot e_{Gd - 148}$$

Q_Gd-148_ in turn can be expressed through either Eqs. (3) or (4) by relating it to the corresponding whole-body activities of ^146^Gd and ^153^Gd, respectively. The cumulative committed effective dose as a function of time per a chronic daily intake of I_Gd-148_ (Bq day^−1^) can then be deduced by the following:

11 $$E_{Gd - 148} \left( t \right) = e_{Gd - 148} \cdot \left( {k_{148/146} \left( t \right) \cdot Q_{Gd - 146} \left( t \right)} \right)/q_{Gd - 148} \left( t \right)) \cdot \,t$$

or

12 $$E_{Gd - 148} \left( t \right) = e_{Gd - 148} \cdot \left( {k_{148/153} \left( t \right) \cdot Q_{Gd - 153} \left( t \right)/q_{Gd - 148} \left( t \right)} \right) \cdot \;t$$

Hence, by numerically computing the time-dependent ratios q_Gd-148_, k_148/146_(t), and k_148/153_(t) using the ICRP models ICRP 100^14^, ICRP 119^7^, and ICRP 141^12^, the whole-body activity and associated cumulative committed effective dose from the alpha emitter ^148^Gd can be related to the measurable quantities Q_Gd-146_ or Q_Gd-153_ for a given set of isotope release ratios, a_148/146_ and a_148/153_, respectively.

### Inhalation of radiogadolinium

Acute intakes through inhalation can lead to significant proportions of systemic activities of ^148^Gd, even after full excretion of the initial GI contents. It is assumed that inhalation of radiogadolinium is only relevant during the immediate phase after a release event. A varying amount of the inhaled ^148^Gd will then be taken up into the systemic tissues depending on the absorption rate from respiratory tract to blood (ICRP^12^). If inhaled in oxide form, most of the gadolinium will be confined to the lungs, even months after inhalation. However, when considering the total body burden of ^148^Gd, Q_Gd-148_ (Bq), for an acute inhalation of ^148^Gd, I_inh,Gd-148_ (Bq), the measured body burdens of ^146^Gd or ^153^Gd at time t after intake can then be expressed as

13 $$Q_{Gd - 146} \left( t \right) = I_{inh,Gd - 148} \cdot a_{146/148} \left( {t = 0} \right) \cdot R\left( t \right);{ }Q_{Gd - 153} \left( t \right) = I_{inh} \cdot a_{153/148} \left( {t = 0} \right) \cdot R\left( t \right)$$

where R(t) is the retention curve for ^146^Gd (N.B. not decay corrected) upon inhalation of the radiogadolinium. To our knowledge, it is not well-known which particle diameter should be expected in different accident scenarios^13^. Given the lack of this knowledge, here we use the retention derived from the ICRP model^12^, with inhalation parameters s_b_ (= 0.021 day^−1^), s_r_ (= 0.3 day^−1^), s_s_ (= 0.002 day^−1^), f_r_ (= 0.5), and f_b_ (= 0.07), which are essentially based on a human volunteer study on inhalation of ^153^Gd_2_O_3_ particles in 2002^19^. The parameters correspond to a moderate rate of absorption (Type M) and to an activity median aerodynamic diameter (AMAD) particle size of 2.2 μm (ICRP^12^).

Particle size is an important parameter affecting the dose calculations. The Swedish Radiation Safety Authority uses an AMAD of 1 μm in their dispersion and dose calculations for the boundary accident scenario (smaller particle sizes are not applicable in the dispersion model used by SSM) and has performed a sensitivity analysis for particles with an AMAD > 5 μm^3^. According to the bioassay software tool, IMBA (Integrated Modules for Bioassay Analysis^20^), the particle size assumed here will yield a committed effective dose of 1.26·10^–5^ Sv per unit inhaled Bq ^148^Gd, which is about a factor of two less than that for a Class F (fast absorption rate) particle in the size range 1 to 5 μm but somewhat higher than the corresponding values for M Class particles in the same size range. The corresponding effective doses for ^146^Gd and ^153^Gd are orders of magnitude lower: 7.6 and 2.5 nSv Bq^−1^, respectively.

Hence, I_inh,Gd-148_ can be deduced from a_146/148_, the R(t) function, and the measured whole-body burden of the gamma-emitting ^146^Gd or ^153^Gd. The corresponding committed effective dose from ^148^Gd will then be

14 $$E_{Gd - 148} = I_{inh,Gd - 148} \cdot e_{Gd - 148,inh} = \left( {Q_{Gd - 146} /a_{146/148} \left( {t = 0} \right) \cdot R\left( t \right)} \right) \cdot e_{Gd - 148,inh} = \left( {Q_{Gd - 146} /a_{153/148} \left( {t = 0} \right) \cdot R\left( t \right)} \right) \cdot e_{Gd - 148,inh}$$

where e_Gd-148,inh_ is the dose coefficient computed by the software IMBA, given the retention functions and associated parameters mentioned previously. Thus, the committed effective dose, E_Gd-148_, from an acute inhalation of ^148^Gd could be estimated through a whole-body burden measurement of ^146^Gd or ^153^Gd.

### Target and release activity ratios of ^146^Gd, ^148^Gd, and ^153^Gd

Activity ratios a_146/148_ and a_153/148_ were evaluated using data obtained from simplified ESS target modeling of the radionuclide composition [2new]. All major components of the ESS target were included in the model with simplified geometries. The FLUKA code was used for calculations, as it is suitable for calculations of particle transport and interactions with matter using the Monte Carlo method^17,18^. We obtained about a factor of 2 higher absolute values of ^148^Gd in comparison with other authors^21,22^, and these differences can be attributed to differences in spallation and nuclide evaporation models. Unfortunately, there are no experimental data yet to evaluate which of the predictions is more accurate regarding absolute values. Activity ratios a_146/148_ and a_153/148_ were calculated for different operation times and decay periods, up to 350 days after 5 years of target operation (designed lifetime of the target).

### Estimating ^148^Gd whole-body burden and cumulative committed effective dose by means of high-resolution gamma spectrometry

In combination with estimated activity ratios of ^146^Gd, ^148^Gd, and ^153^Gd in the spallation target and the biokinetic models described in Eqs. (7) and (13), the minimum detectable activity (MDA) of the alpha emitter ^148^Gd for a high-resolution whole-body counting system, consisting of a 123% high purity germanium (HPGe) described by Rääf et al.^8^, was calculated. The whole-body counter is calibrated for a uniform body distribution of gamma emitters, but in this study alternative uptake geometries were needed to better mimic the anticipated uptakes of subjects exposed to internal radiogadolinium contamination. Using the VMC in vivo tool (VMC 2018^23^), the relative difference in the efficiency calibration of a HPGe whole-body counter between a uniform whole-body distribution of gamma emitters in the energy range 100 to 150 keV, and that of specific organ uptakes could be simulated. In this tool the geometry of lung uptake in male adult phantom was available and used here for acute inhalation of a gamma emitter, whereas an uptake in the liver in the same phantom was used to mimic the calibration factor for a whole-body counting with elevated uptake in the abdominal region. The calibration factors for the 123% HPGe system in the photon energy range of 100 to 150 keV (roughly encompassing the considered gamma lines of ^146^Gd and ^153^Gd given in Table 1) could then be corrected by a factor of 2 (± 10% k = 1) for abdominal region uptake and by a factor of 0.66 (± 10% k = 1) for lung uptake. The MDA_Gd-148_ value in combination with Eq. (14) could then be used to estimate the corresponding minimum detectable committed effective dose, MDD_Gd-148_. The MDA and MDD values as a function of time of the after the release, for two different operation times (1 and 5 y) were explored. Finally, the potential perturbations from other gamma lines present will be discussed, based on simulations of gamma spectra.

## Results and discussion

### Simulated relative W-target inventories of radiogadolinium and assumed daily ingestion after a release

**Figure 2:**
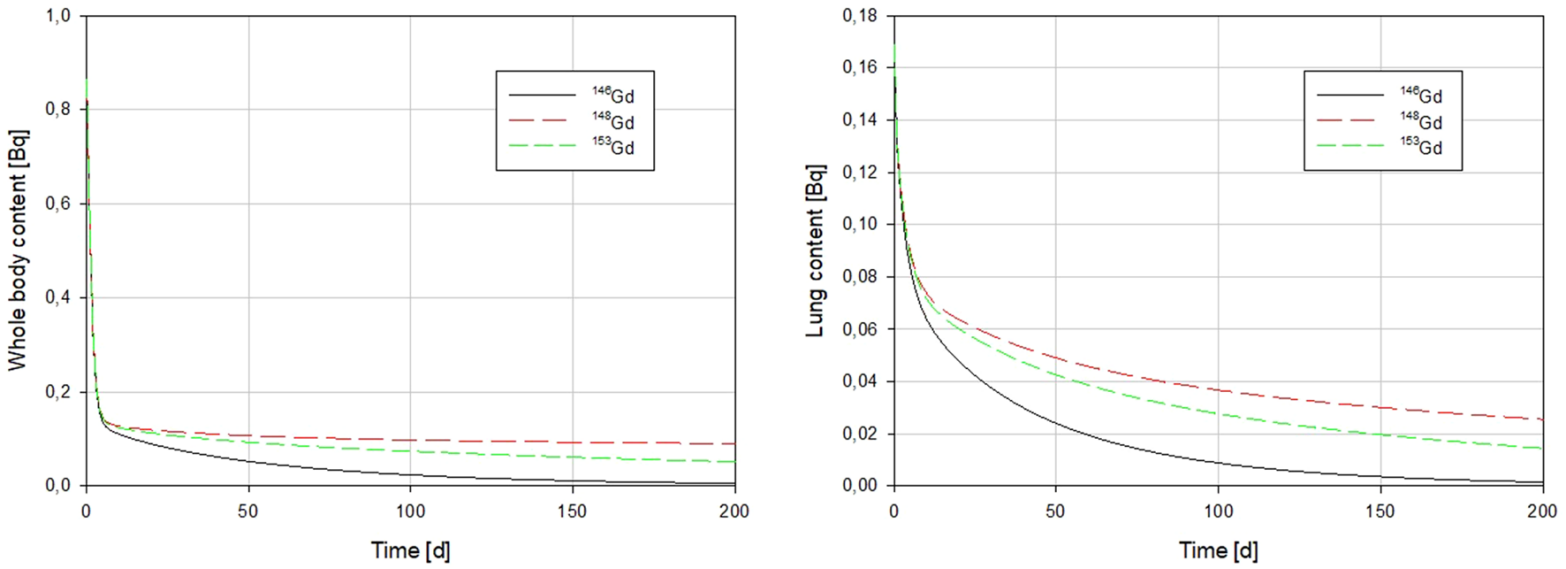
Isotope specific retention curves, R(t), for ^146^Gd, ^148^Gd, and ^153^Gd upon inhalation. Left: Whole body. Right: Lung model taken from ICRP^12^ using f_b_ = 0.07, f_r_ = 0.5, s_b_ = 0.021 d^−1^, s_s_ = 0.002 d^−1^, and s_r_ = 0.3 d^−1^. Parameters are further explained in ICRP 130^24^.

Simulated W-target activity ratios between ^146^Gd and ^148^Gd and between ^153^Gd and ^148^Gd, respectively, during operation of the ESS target are given in Fig. 2 (left). The corresponding activity ratios for dispersed W-target particles as a function of time after the release are plotted in Fig. 2 (right). The activity ratio values taken from the ESS Preliminary Safety Analysis Report (PSAR)^22^ and SSM report on emergency preparedness planning around the facility^3^ are also provided in Fig. 2. The ratios from those reports are higher, i.e., they predict relatively lower activities of ^148^Gd in comparison with other gadolinium isotopes. Our predictions might be considered more conservative in terms of relative proportion of the alfa emitting gadolinium isotope, but experimental data are necessary to prove this hypothesis. The SSM report^3^ also suggests that ^148^Gd deposition on the ground might be monitored using the gamma-emitting ^146^Gd, considering the activity ratio of these radionuclides.

**Figure 3:**
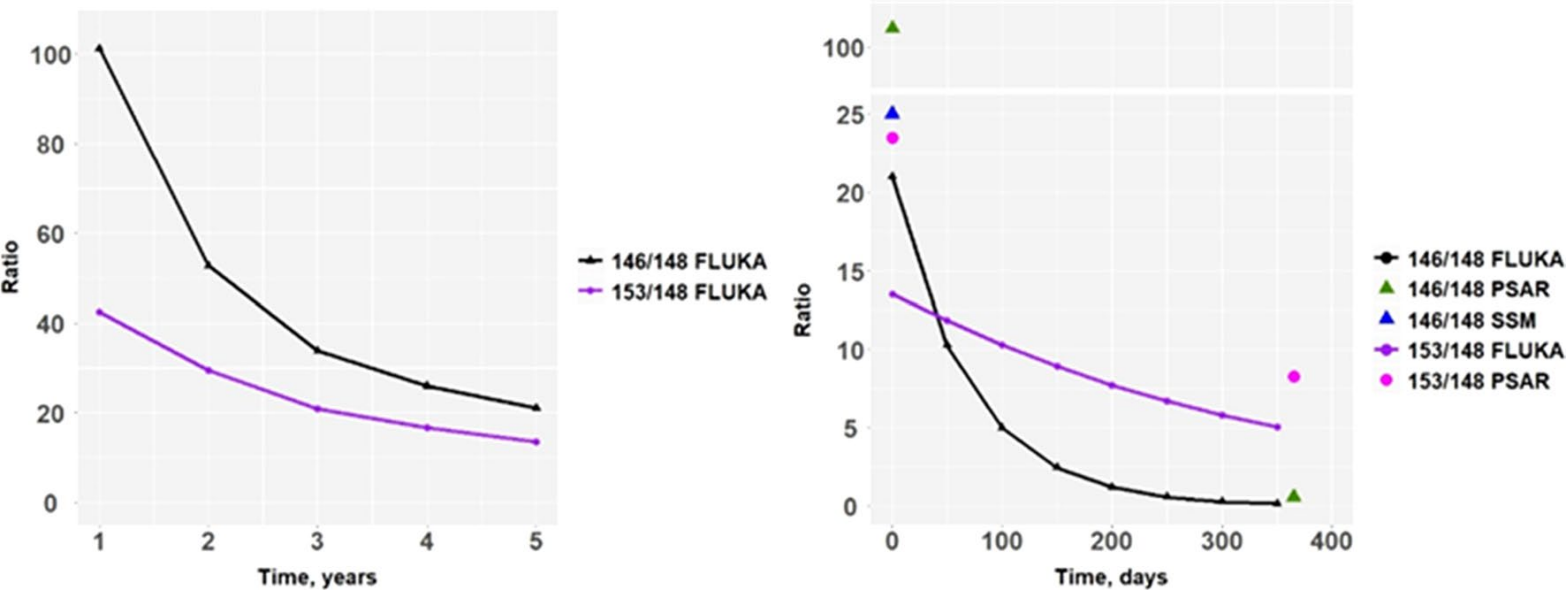
Left: Modeled activity ratios of ^146^Gd to ^148^Gd (a_146/148_) and ^153^Gd to ^148^Gd (a_153/148_) in the W target as a function of operation time. These values correspond to a_146/148_(t_0_) and a_153/148_(t_0_) in Eqs. 12 and 13. Right: Activity ratios in a W target after 1 y operation as a function of time after the cessation of operation. Values are simulated using FLUKA. Numbers from ESS PSAR (2012)^22^ and SSM report^3^ are provided for comparison.

Note that the abovementioned activity ratios will represent the initial release activity ratios, a_146/148_ and a_153/148_, in the case of an accidental atmospheric release either during or after operation. The resulting daily ingestion of ^146^Gd and ^153^Gd normalized to that of ^148^Gd, assuming only physical decay in the environment, is given in Fig. 3 for a number of different target operation times (1 to 5 y).

### Body burdens of ^148^Gd as a function of time relative to that of ^146^Gd and ^153^Gd and its dosimetric effect

**Figure 4:**
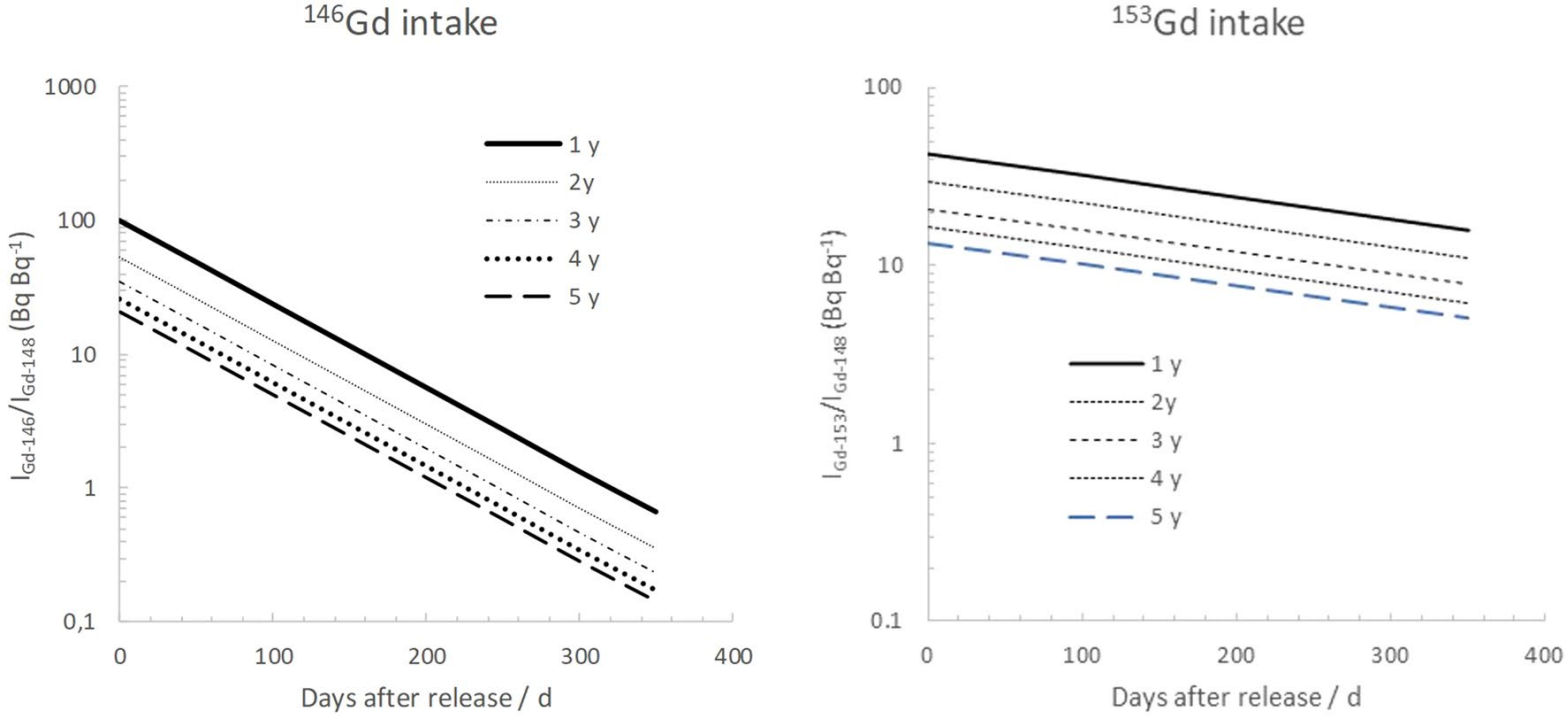
Simulated activity ratios of daily assumed ingestion of ^146^Gd (left) and ^153^Gd (right), normalized to that of ^148^Gd, I_Gd-148_, as a function of time after an environmental release of particles from the W target. Values are based on simulated activity ratios for different operation times of the ESS tungsten target.

Based on the FLUKA simulations of the activity ratios in the target, values of a_146/148_ and a_153/148_ in adult individuals subjected to a protracted intake of environmentally dispersed target material can be estimated. From these values, the resulting proportions (k_148/146_ and k_148/153_) between the whole-body activity of ^148^Gd and the gamma emitters ^146^Gd and ^153^Gd can be computed for a 1 to 5 years operation time (Fig. 4). It can be seen that, after 1 year of continuous intake of gadolinium isotopes released from a 5 years operation W spallation target, the model predicts a body content of 8.8 Bq of ^148^Gd for every Bq of ^146^Gd in an adult person. The corresponding value for ^153^Gd is much less, only a value of 0.21 (Bq Bq^−1^). The longer physical half-time of ^153^Gd will outweigh its lower initial isotopic abundance in the aforementioned release event, and after about 40 days after a release from a 5 years operated W target, the body content of ^153^Gd will be higher than that of ^146^Gd.

**Figure 5:**
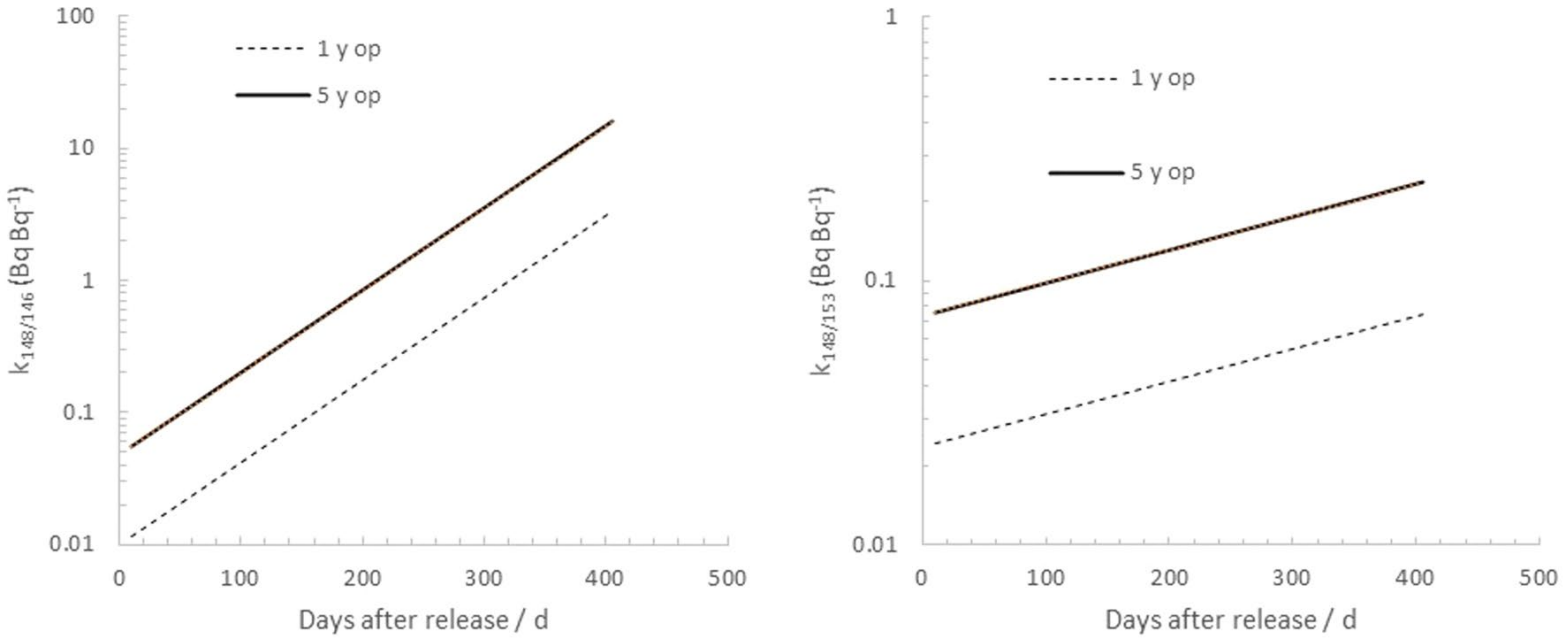
Left: The ratio between whole-body activity of ^146^Gd and ^148^Gd as a function of time after onset of chronic intake of 1 Bq d^-1^ of ^148^Gd after a release from a tungsten target after 1 and 5 y of operation. Right: The same plot for ^153^Gd.

Figure 5 plots the corresponding cumulative committed effective dose as a function of time per unit whole-body activity of ^146^Gd and ^153^Gd, respectively, assuming a daily intake, I_Gd-148_, of 1 Bq day^−1^. From these plots, it can be seen that the model predicts a cumulative committed effective dose of 0.30 μSv from ^148^Gd per detected activity (Bq) of ^146^Gd in the whole body, if observed 1 years post release of the W target (5 years operation). For ^153^Gd, this value is considerably lower: 7.1 nSv per unit observed whole-body activity (Bq) of ^153^Gd. This implies that the detection of ^153^Gd in vivo will, in theory, be a much more sensitive indicator of ^148^Gd cumulative committed effective dose than ^146^Gd when surveying potentially affected persons, already one-month post release from the W target.

**Figure 6:**
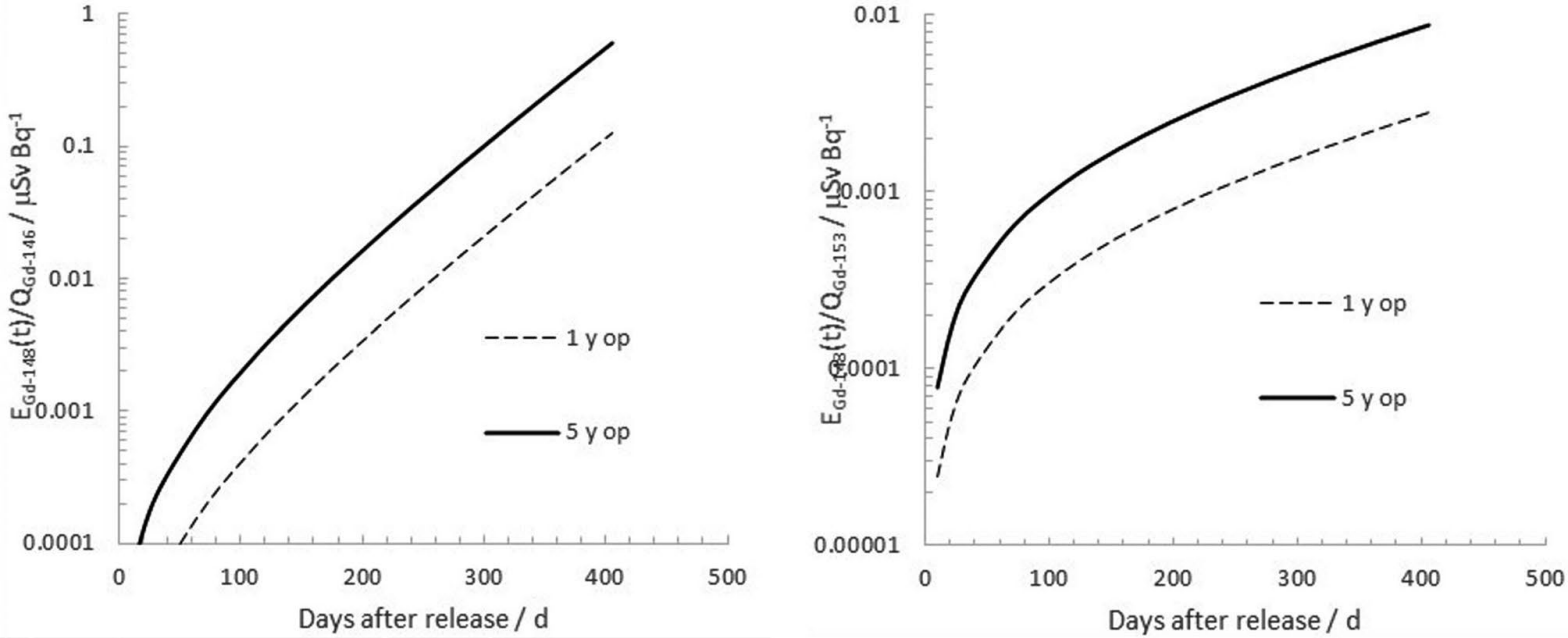
Left: Cumulative committed effective dose from ^148^Gd per measured whole-body burden of ^146^Gd, E_Gd-148_(t)/Q_Gd-146_, as a function of time after onset of chronic ingestion of 1 Bq d^-1^ of ^148^Gd. Right: The same plot for ^153^Gd.

The relative contributions to the cumulative committed effective dose from ^146,153^Gd and ^148^Gd are given in Fig. 6. Only after some months after the start of the protracted radiogadolinium ingestion does the alpha emitter ^148^Gd account for the larger part of the cumulative committed effective dose from the three major gadolinium isotopes. One year after the onset of the ingestion, the radionuclide will account for 89% of the cumulative effective dose incurred from the three major gadolinium isotopes for an adult.

### Detection limits of whole-body burden and cumulative committed effective dose of radiogadolinium isotopes for a high-resolution whole-body counting system

For the 123% HPGe detector setup described by Rääf et al.^8^ with a pulse acquisition time of 2400 s in a Palmer geometry, the estimated minimum detectable activity, MDA, of ^146^Gd using the 114 + 115 keV and 154.6 keV gamma lines, and a correction factor for enhanced detectability in the abdominal region by a factor of 2.1 described previously, is estimated to be 6.3 and 12 Bq, respectively, for a homogeneous nuclide distribution in the abdominal region for a 70 kg person. For an activity ratio a_146/148_(t) of 21.0 (5 years operation tungsten target) at t = 0, this will give an MDA of 0.31 (using the 115.4 keV peak) and 0.58 Bq (using the 154.6 keV peak) for ^148^Gd. The corresponding minimum detectable committed effective dose, MDD(t) = e_Gd-148_·t·MDA(t)/q_Gd-148_(t), is 0.017 µSv and 0.032 µSv, respectively, for an acute ingestion just 1 day after the release (Table 2). As the amount of ^148^Gd is initially cumulated in the body according to Eq. (3), the detection level will decrease slightly with time; however, within one week, the physical decay of the tracing nuclide ^146^Gd will instead lead to an exponentially increasing detection limit. Hence, the corresponding MDA and MDD values (when using the 115.4 keV peak of the ^146^Gd isotope) for chronically exposed adults will become 56 Bq and 810 μSv after 1 year post release of a 5 years operated W particle release (Table 2).

**Table 2:** Theoretical minimum detectable whole-body activity (MDA) and corresponding minimum detectable committed effective dose (MDD) using either the 115.4 keV or 154 keV photo peaks of ^146^Gd as a marker for the whole-body activity of ^148^Gd for chronic ingestion and for an acute inhalation for an adult male (AMAD = 2.2 μm).

Time after release at t_0_	*MDA*_*Gd-148*_ (Bq)				*MDD*_*Gd-148*_ (μSv) Chronic ingestion				*MDA*_*Gd-148,inh*_ (Bq) Acute inhalation at t_0_				*MDD*_*Gd-148,inh*_ (μSv) Acute inhalation at t_0_			
	1 y op		5 y op		1 y op		5 y op		1 y op		5 y op		1 y op		5 y op	
	115.4 keV	154.6 keV	115.4 keV	154.6 keV	115.4 keV	154.6 keV	115.4 keV	154.6 keV	115.4 keV	154.6 keV	115.4 keV	154.6 keV	115.4 keV	154.6 keV	115.4 keV	154.6 keV
1 d	0.0634	0.121	0.306	0.582	0.0035	0.0067	0.0168	0.032	0.362	0.689	1.74	3.31	4.57	8.68	22.0	41.8
7 d	0.069	0.132	0.334	0.635	0.0178	0.0339	0.0857	0.163	1.68	3.19	8.08	15.4	21.7	40.2	102	194
30 d	0.096	0.183	0.464	0.882	0.107	0.203	0.513	0.975	2.73	5.19	13.18	25.0	34.4	65.4	165	315
1 y	11.6	22.2	56.0	107	168	320	808	1540	481	915	2310	4400	6060	11,500	29,100	55,400

If instead ^153^Gd (with either the gamma lines at 97.4 and 103.2 keV, respectively) is used as an indicator of body activity and cumulative committed effective dose of ^148^Gd, the detection levels are initially higher than when using ^146^Gd due to the relatively lower initial isotope ratio in the released W-target material (e.g., 42.5 vs. 101 for a target under 1 year operation). As mentioned previously, however, 1 year post release it is evident that ^153^Gd will be a much more sensitive indicator for the presence of ^148^Gd, with significantly lower MDA and MDD values compared with ^146^Gd (Table 3).

**Table 3:** Theoretical minimum detectable whole-body activity (MDA) and corresponding minimum detectable cumulated dose (MDD) using either the 97.4 keV or 103.2 keV photo peaks of ^153^Gd as a marker for the whole-body activity of ^148^Gd for chronic ingestion and for an acute inhalation for an adult male (AMAD = 2.2 μm).

Time after release at t_0_	*MDA*_*Gd-148*_ (Bq)				*MDD*_*Gd-148*_ (μSv) Chronic ingestion				*MDA*_*Gd-148.inh*_ (Bq) Acute inhalation t_0_ = 0				*MDD*_*Gd-148.inh*_ (μSv) Acute inhalation at t_0_			
	97.4 keV	103.2 keV	97.44 keV	103.2 keV	97.4 keV	103.2 keV	97.4 keV	103.2 keV	97.4 keV	103.2 keV	97.4 keV	103.2 keV	97.4 keV	103.2 keV	97.4 keV	103.2 keV
1 day	0.443	0.585	1.40	1.85	0.0243	0.0328	0.0767	0.102	2.52	3.33	7.93	10.5	31.8	42.0	100	132
7 day	0.450	0.596	1.42	1.88	0.116	0.153	0.365	0.482	10.9	14.4	34.3	45.4	137	182	432	572
30 day	0.481	0.639	1.52	2.01	0.532	0.704	1.68	2.22	13.6	18.0	42.7	56.6	171	226	539	713
1 year	1.27	1.68	4.01	5.31	18.4	24.3	57.9	76.6	50.9	67.4	160	212	642	849	2020	2670

**Figure 7:**
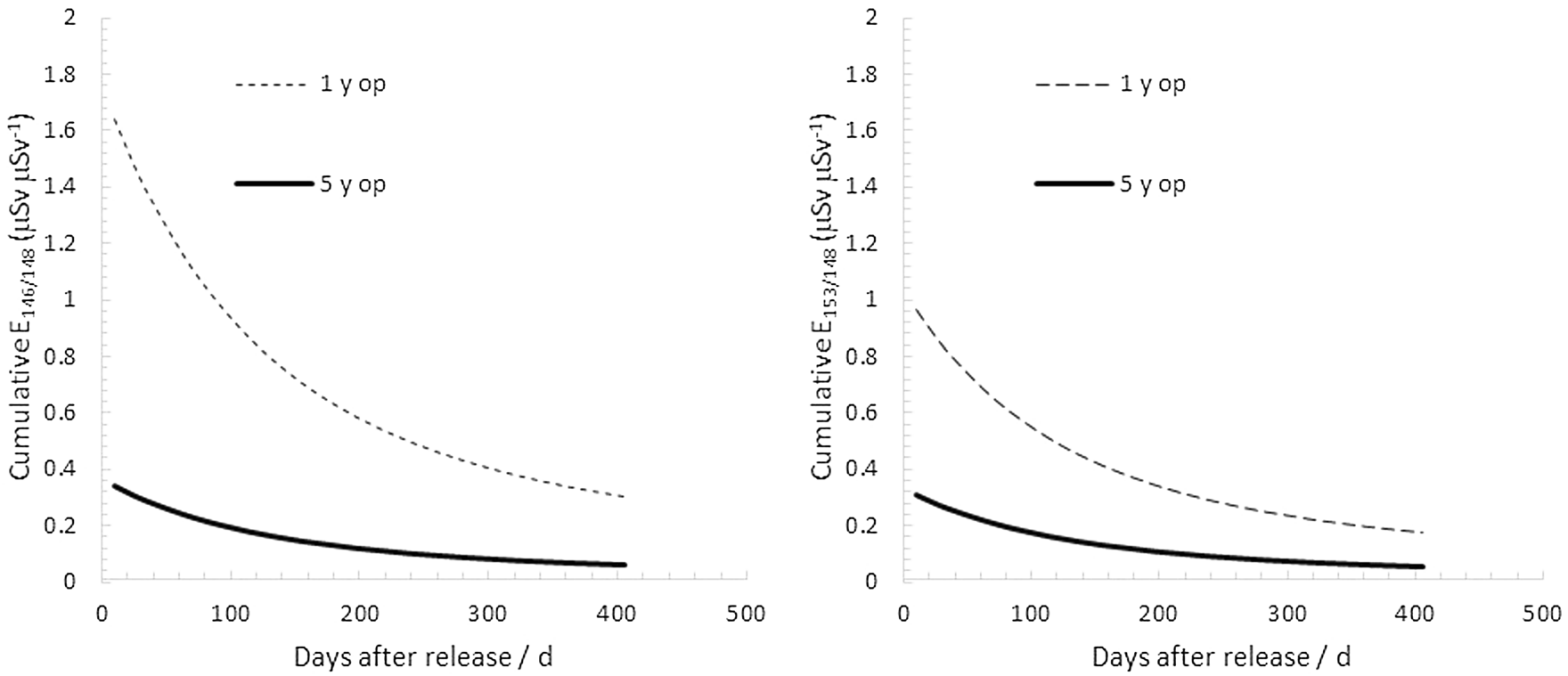
Left: The ratio between the cumulative committed effective dose from 146Gd and from 148Gd as a function of time after onset of chronic ingestion of 1 Bq d^-1^ of ^148^Gd. Right: The same plot for ^153^Gd.

For gadolinium in oxide form, up to 50% of inhaled radiogadolinium will be accumulated in the lungs (^12^; see also Fig. 7), and the measurement geometry in vivo would therefore be a torso geometry, as previously mentioned in the Section “Estimating ^148^Gd whole-body burden and cumulative committed effective dose by means of high-resolution gamma spectrometry”. This gives rise to a corresponding factor of 3.2 increase in MDA of the primary photon peaks in this energy region of ^146^Gd and ^153^Gd, compared with assuming an abdominal uptake, and thus a corresponding increase in the indirect determination of ^148^Gd. From the results given in Table 2, it can be seen that MDD values can be reasonably low (< 0.20 mSv) using high-resolution whole-body counting of ^146^Gd as a trace nuclide for the internal dose of ^148^Gd if measured within 30 days upon release, regardless of whether the uptake occurred through ingestion or inhalation. However, for longer monitoring delays, it appears that ^153^Gd will be a much more sensitive indicator of inhaled ^148^Gd, regardless of the operation history of a W target before release. Nevertheless, it will then not be plausible to determine committed effective doses from acute inhalations lower than about 3 mSv, even if using ^153^Gd (Table 3).

### Perturbations in whole-body gamma spectrometry of radiogadolinium

**Figure 8:**
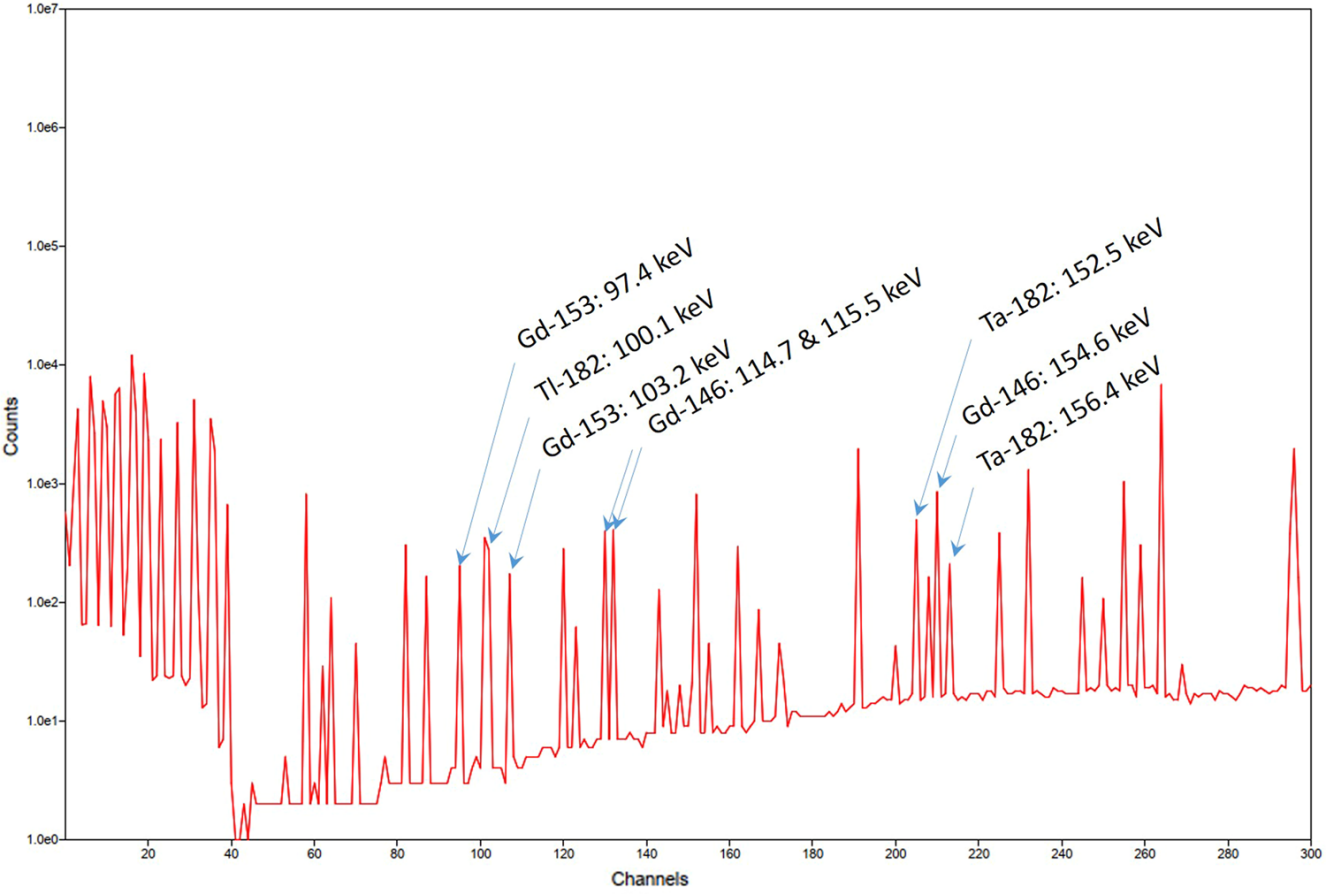
Simulated spectrum from a W spallation target, operated for 5 years. The emission spectrum refers to 50 days post end of operation.

In addition to the theoretical detection limits, the presence of perturbing radionuclides must also be considered. A representative W-target particle was investigated that contained a radionuclide composition according to our calculations^2^. Monte Carlo N-Particle (MCNP) code simulations^25^ of the emission spectrum from a representative W-target particle are shown in Fig. 8. It appears that a number of perturbing gamma emitters will be present, of which the ^182^Ta (t_½_ = 114.4 d) peaks at 152.5 keV (n_g_ = 0.070) and 156.4 keV (n_g_ = 0.027) will most definitely affect the 154.6 keV line of ^146^Gd. Tantalum has uptake properties similar to those of gadolinium (ICRP 119), and inhalation or ingestion of gadolinium may be accompanied with corresponding intakes of ^182^Ta. Ongoing work will shed light on the time window for in vivo determination of inhaled ^146^Gd in lungs and of the various potential contributions to the internal dose from spallation source products.

## Conclusions

A potential release of W-target material from a spallation source may lead to atmospheric dispersion of radioactive gadolinium which is continuously generated in the target during the spallation operation. According to ICRP, the predominant effective dose contribution of the gadolinium isotopes will be from ^148^Gd due to its alpha emission and can in an accident scenario with atmospheric dispersion of the nuclides potentially lead to significant internal exposures through inhalation. A theoretical investigation has been done of a method to determine internal exposures from inhaled or ingested ^148^Gd in affected subjects using in vivo whole-body counting in combination with pre-calculated activity ratios between the alpha emitter ^148^Gd and the corresponding gamma-emitting gadolinium isotopes ^146^Gd and ^153^Gd. ^146^Gd will initially be the most sensitive indicator of the ^148^Gd internal dose, but some months after a release event, ^153^Gd will, in theory, be a much more sensitive ^148^Gd dose indicator. For a 123% HPGe detector used in Palmer geometry, 1-year post release, in vivo detection of ^153^Gd can yield a minimum detectable cumulative committed effective dose from ^148^Gd ranging from 18 to 77 μSv for ingested ^148^Gd, and 0.64 to 2.7 mSv for acutely inhaled ^148^Gd, depending on the operational age of the released spallation target material and on which gamma peak (97.4 or 103.2 keV) is used in the assessment. However, preliminary Monte Carlo simulations of particle emission spectra from a W target in a spallation source being operated for 5 years show that the ^182^Ta peak may potentially perturb some of the investigated primary gamma lines from ^146^Gd and ^153^Gd. If that is the case, in vivo detection of gadolinium uptake can be made indirectly through the ^146^Gd daughter, ^146^Eu. This is to be investigated further in continued studies.
